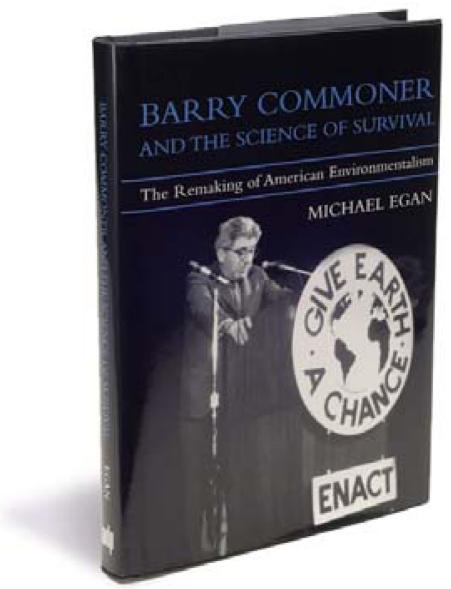# Barry Commoner and the Science of Survival: The Remaking of American Environmentalism

**Published:** 2007-11

**Authors:** Peter R. Jutro

**Affiliations:** Peter R. Jutro received his PhD from Cornell, where he subsequently taught and did research in epidemiology, risk assessment, and environmental policy. He served as a science advisor in Congress specializing in water and disaster issues, and is now Deputy Director for Science and Policy of the U.S. Environmental Protection Agency’s National Homeland Security Research Center

By Michael Egan

Cambridge, MA:MIT Press, 2007. 283 pp. ISBN: 978-0-262-05086-9, $28

These days, no one scientist is so intimately associated in the general public mind with a particular scientifically based social problem as Barry Commoner was with the growth of the environmental movement in the mid-20th century. Commoner is a key, underexamined figure well worth introducing to the current generation of scientists and environmentalists, and in this densely referenced book, Michael Egan takes on the challenge.

The book begins with an analysis of the post–World War II consequences of American technological optimism, moves to the mid-century debate over nuclear fallout and the role of technical information, reviews Commoner’s role in ecology, explores the population-versus-technology debates over the origin of environmental degradation, examines the oil crisis of the 1970s and its relation of risk to economics, and finally discusses the relationship between poverty and environmental risk.

Although the structure of the book is initially explained, it results in considerable repetition of fact and analysis. That, together with annoying factual errors, such as putting the Trinity site of the first atomic explosion at Los Alamos, New Mexico, instead of at the White Sands Proving Ground, more than 200 miles south, or contradictory references to restoring the integrity of science and preserving the integrity of science, leads a reader to feel that this serious, scholarly work would have benefited from more intensive attention from its editors. Any structural faults aside, the book capably illuminates the sweep of Commoner’s involvement in social issues of the last half-century, and makes a major contribution to the literature on the origins of current environmental debates.

At a time when it was not the common wisdom, Commoner firmly believed that an informed public was the key to intelligent governmental decision making, and that the duty of a scientist was to inform the public. However, he also believed that free-market capitalism, which he found socially irresponsible, was, as Egan writes, “a clear enemy”; Commoner’s leftist background yielded analyses congruent with a “holistic critique of American social structures.”

Although Commoner’s career ranged from bench plant physiology at Washington University to social and environmental activist to 1980 presidential candidate, he may be best known for his “Four Laws of Ecology”: “everything is connected to everything else”; “everything must go somewhere”; “nature knows best”; and “there is no such thing as a free lunch.” These “laws” in their straightforward simplicity are brilliantly accurate, descriptive, and evocative. Coupled, however, with Commoner’s advocacy of the precautionary principle—a moral and political concept that evolved from the German democratic–socialist legal tradition of the 1930s—they can be seen as forcing stalemate, his laws warning society to proceed with care, but the precautionary principle virtually eliminating any possible path along which to proceed.

Where Commoner visualized risk assessment as an essential analytical and informational tool for implementing the precautionary principle, over time the two have evolved into competing, politically driven approaches. This competition can be of considerable economic consequence, perhaps best seen in recent disputes between the United States and the European Union over the regulation of genetically modified organisms.

Although perhaps not Egan’s intent, the book also uses Commoner’s career to help the reader gain insight into fascinating elements of the evolution of the involvement of the American Association for the Advancement of Science in the social politics of environmental science, and to illuminate key portions of the early radiation-based history of environmental risk assessment.

Commoner emerged as a public figure with the nuclear fallout issue of the 1950s and 1960s. What this portion of the narrative suggested to me was that Commoner’s view of the conflict between national security and public health should be reformulated. Maintenance of an economically sound, healthy population and environment must be thought of as key elements of national security, rather than as its competitors.

In the end, we get from Egan an analytical, reasoned picture of Commoner—clearly a seminal figure in the history of American environmentalism—and of his role in that environmentalism. Commoner’s background, biases, aspirations, and intentions are well described and intriguingly tied to analyses of his activities. In the process, we also learn about many intertwined political movements of the last half-century, and from this we get a clearer picture of the evolution of American and international environmental politics.

## Figures and Tables

**Figure:**